# STG: Structured Topology of Gridpoints for Occluded Pedestrian Detection

**DOI:** 10.3390/s26175496

**Published:** 2026-08-30

**Authors:** Tian Qiu, Jifeng Shen, Xin Zuo

**Affiliations:** 1 School of Electrical and Information Engineering, Jiangsu University, Zhenjiang 212013, China; 3230502011@stmail.ujs.edu.cn; 2 School of Computer Science and Engineering, Jiangsu University of Science and Technology, Zhenjiang 212003, China; zuoxin@just.edu.cn

**Keywords:** occlusion handling, pedestrian detection, gridpoints modeling, transformer, feature interaction

## Abstract

Pedestrian detection in crowds is a challenging problem in computer vision. Existing occlusion-handling methods heavily rely on expensive visible-box annotations to locate visible body parts, posing severe limitations in label acquisition cost and open-world generalization. To break through this limitation, we propose a novel Structured Topology of Gridpoints (STG) framework. Operating strictly under standard full-box annotations without any extra visibility supervision, STG aims to achieve implicit, fine-grained local semantic compensation. Specifically, we formulate a coarse-to-fine reasoning paradigm consisting of three interactive stages. To mitigate the high spatial complexity and eliminate background redundancy, we first introduce a Saliency-Aware Feature Filtering (SAFF) mechanism, which leverages gridpoint heatmaps to filter out low-confidence pedestrian candidates. Second, a query-guided Across-Instance Feature Interaction (AIFI) model is designed to utilize inter-instance spatial relationships to propagate missing context from highly visible individuals to their occluded neighbors. Finally, we devise a prior-guided Inner-Instance Gridpoints Interaction (I2GI) model to achieve fine-grained structured part-level feature completion, which dynamically aggregates vital localized cues from diverse human parts to reconstruct holistic pedestrian representations. Extensive experiments on the CityPersons, CrowdHuman, and WiderPerson datasets demonstrate the effectiveness and efficiency of our proposed method. Specifically, STG achieves a log-average miss rate of 7.41% on Reasonable and 32.05% on Heavy Occlusion subsets of CityPersons, while running at up to 16 FPS, outperforming existing part-based methods under full-box supervision.

## 1. Introduction

Pedestrian detection is an important research topic in the field of computer vision, with downstream applications such as video surveillance, action recognition, and autonomous driving. In recent years, there has been a lot of excellent research [1,2,3] to advance this task. Pedestrian detection methods are generally guided by generic object detection research [4,5,6,7], thanks to the flourishing of convolutional neural networks [8,9,10]. As tasks in these areas often require timely decisions, pedestrian detectors are generally expected to be not only accurate but also efficient. In addition, landing pedestrian detection systems may encounter extra constraints such as lower computational complexity.

Although recent years have witnessed remarkable advances in pedestrian detection, occlusion still poses a critical bottleneck for real-world deployments. To tackle this challenge, the key lies in learning robust feature representations that can isolate human cues from surrounding interference. Conventional methods [8,11] concentrate on the acquisition of semantic features related to pedestrians from the entire bounding box. These methods typically rely on holistic pedestrian representations while overlooking the substantial background noise encapsulated within the bounding box under occlusion. Consequently, these methods suffer significant performance degradation due to their inability to discriminate more finely the interference information within the bounding box in densely populated settings. Additonally, recent studies employ part-based methods to divide the bounding box into multiple regions or specific body parts. For instance, while earlier works [12,13] rely on manual sub-region partition, recent advanced frameworks like OADB-Net [14] utilize dual-branch architectures to couple full-body and head-shoulder regions, and MPLR-CapsNet [15] employs multi-line parallel features with capsule reasoning to infer occluded attributes. By leveraging extra annotations or advanced part-level reasoning, these methods successfully guide the detector to distinguish heavily occluded instances, thereby proving effective in mitigating performance degradation.

**Figure 1:**
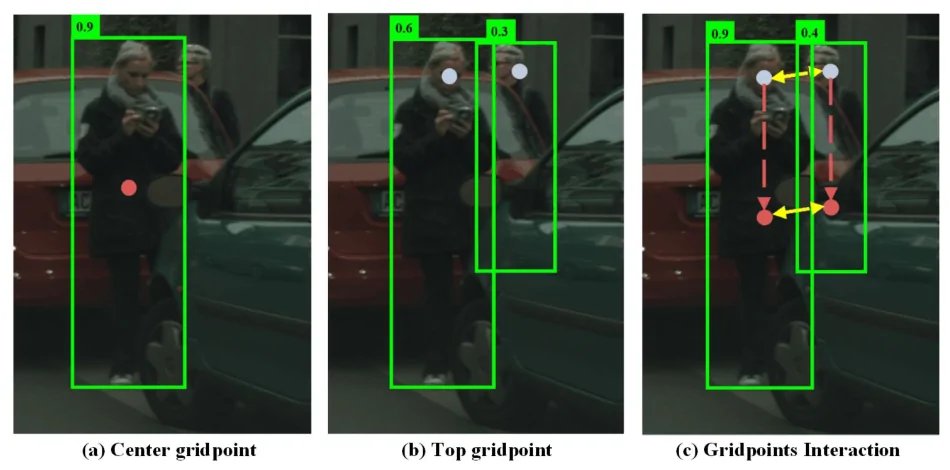
Overview of the STG framework for pedestrian detection under occlusion. Red and blue dots represent gridpoint proposals detected by the Center branch (**a**) and Top branch (**b**), respectively. (**c**) Yellow arrows indicate across-instance context propagation via the AIFI module, transferring structural cues from an unoccluded pedestrian to a heavily occluded neighbor. Pink arrows denote inner-instance feature aggregation via the I2GI module, completing missing local features across different anatomical body parts within the same individual.

To address these limitations under standard full-box supervision, we propose a novel Structured Topology of Gridpoints (STG) framework for crowded pedestrian detection (Figure 1). Rather than relying on coarse bounding boxes, STG represents pedestrians through a set of discrete, flexible gridpoints to enable fine-grained feature decoupling. To systematically tackle holistic feature degradation under occlusion while avoiding quadratic computational growth, we organize our solution into a cohesive three-tiered architecture:

The first tier is **Foreground Feature Filtering via SAFF**. To prevent high computational complexity when interacting all candidate gridpoints across an image, we introduce the SAFF module. SAFF dynamically identifies high-response foreground regions from heatmaps, efficiently isolating informative gridpoint features within the STG from background clutter.

The second tier is **Across-Instance Context Propagation via AIFI**. Detecting heavily occluded instances remains inherently variable, often yielding low-confidence predictions due to extreme local corruption. Given that the Transformer architecture excels at modeling non-local feature associations, we first extend our reasoning to the crowd level via the AIFI model. Driven by a sparse self-attention transformer mechanism, AIFI models inter-instance relations by treating visible pedestrians as topological anchors that propagate missing spatial context to heavily occluded neighbors.

The third tier is **Inner-Instance Feature Aggregation via I2GI**. Building upon the cross-instance contextual propagation, a consecutive challenge emerges at the individual level: unlike continuous bounding boxes, gridpoints isolated by STG are intrinsically scattered and decentralized across the body, making their feature interaction difficult. To compensate for missing semantic cues under partial occlusion, the detector must dynamically attend to features at disparate body positions. Consequently, the I2GI model is developed. I2GI employs a topology-guided axial cross-attention mechanism, where feature queries from corrupted local parts cross-attend to unoccluded axial neighbors within the same instance to dynamically complete missing part-level semantics.

To summarize, our main contributions are as follows:

- Structured Topology of Gridpoints (STG): We propose a novel gridpoint topology framework for pedestrian detection. By implicitly embedding anatomical structure into a discrete spatial lattice, STG enables fine-grained part-level modeling strictly under standard full-box supervision, completely eliminating the need for costly visibility annotations or keypoint labels.
- Across-Instance Context Propagation (AIFI): We design an inter-instance interaction module that constructs sparse self-attention among pedestrian queries. This enables heavily occluded targets to leverage unoccluded neighboring pedestrians as structural topological anchors, effectively resolving cross-object feature confusion.
- Inner-instance Axial Feature Compensation (I2GI): We introduce an inner-instance interaction module driven by prior-guided axial cross-attention. By constraining feature aggregation strictly along horizontal and vertical body axes, I2GI dynamically completes corrupted local features using visible anatomical parts while preventing attention dispersion into background noise.

The remainder of this paper is organized as follows. Section 2 provides an overview of related work. Section 3 presents a detailed description of our proposed method. Experimental results are presented in Section 4, and Section 5 concludes the paper.

## 2. Related Work

### 2.1. Pedestrian Detection

Pedestrian detection serves as a foundational component for various applications like autonomous driving, yet it faces distinct challenges such as scale variations, pose diversities, and structural occlusions [16,17,18]. Early deep learning approaches adapted generic detectors to this task through targeted architectural modifications. For instance, RPN+BF [19] combined a Region Proposal Network with a Boosted Forest to address low-resolution and class-imbalance issues, while ALFNet [20] utilized progressive detection heads on SSD [21] to refine initial anchors step-by-step. To bypass hand-crafted anchor design, anchor-free paradigms like CenterNet [22] and CSP [23] formulated pedestrian detection as a center-point and scale prediction task on high-resolution feature maps.

More recently, the landscape of pedestrian detection has expanded into advanced paradigms, including multispectral feature fusion [24], multimodal perception [25], and large language model (LLM) collaboration [26]. Concurrently, query-based end-to-end detectors like DETR [27] and its extensions (e.g., DINO [28], Co-DETR [29], and YOLOv10 [30]) have demonstrated exceptional capabilities through positive-sample enrichment and NMS-free optimizations [31,32].

Nevertheless, these generic advanced frameworks predominantly treat each pedestrian as a holistic object representation. They lack explicit mechanisms to accommodate the unique spatial structures and structural semantics of human bodies, rendering them suboptimal when encountering severe feature corruption. This limitation necessitates more specialized designs tailored for crowded and heavily obscured environments.

### 2.2. Pedestrian Detection in Crowded Scenes

Occlusion remains a primary bottleneck in crowded pedestrian detection. Traditional occlusion-handling approaches can be broadly categorized into three streams. First, loss-based methods introduce specialized constraints to regularize bounding boxes; for instance, Repulsion Loss [2] repels overlapping proposals, while Aggregation Loss [3] pulls proposals closer to their designated targets. Second, part-based methods leverage localized body features to handle partial occlusions. Typical works like Bi-Box [4] and OADB-Net [14] utilize dual-branch architectures to couple full-body and visible/head-shoulder regions, while PedHunter [33] enforces explicit head-part supervision. Similarly, ATT [8] incorporates a visible part-based attention mechanism, and MPLR-CapsNet [15] employs multi-line parallel features with capsule reasoning to infer occluded attributes. Third, NMS-based methods adapt the post-processing phase to dense clusters, either by leveraging feature embeddings [34], incorporating target density dynamic thresholds [5,10], or optimizing through neural networks [35]. However, a major limitation of most part-based methods is their heavy reliance on manual, expensive visible-part annotations to guide the model.

Recently, Transformer-based end-to-end detectors have been introduced to alleviate the reliance on hand-crafted NMS in crowded scenarios. For instance, PED [36] adaptively refines attention positions to visible body parts, while AD-DETR [37] proposes a decoupled cross-attention head to restrict the attention range within unoccluded regions, reducing background noise. Additionally, adversarial feature completion techniques [38] utilize the global transformer context to hallucinate degraded features of unseen parts.

Unlike DETR variants that employ global dense attention, which incurs high computational complexity and risks feature dilution in dense crowds, STG utilizes a sparse gridpoint-driven attention design. Furthermore, compared to unrestricted full-image self-attention that easily aggregates background noise under occlusion, I2GI enforces an axial topological constraint. By restricting cross-attention strictly along horizontal and vertical body axes, I2GI prevents attention dispersion and reliably completes corrupted features using unoccluded anatomical neighbors.

Additionally, our STG framework completely discards the need for extra visible-box annotations. By decomposing each pedestrian into structured intrinsic gridpoints and performing both across-instance and inner-instance topological reasoning, STG achieves fine-grained semantic compensation entirely driven by prior-guided structural knowledge.

### 2.3. Point-Based Object Detection

For a long time, anchor-based frameworks [21,39,40] dominated object detection, yet their reliance on predefined anchors introduces tedious hyperparameters and limits generalization. To overcome these constraints, anchor-free point-based methods have emerged as a prominent alternative. Early paradigms captured targets via boundary keypoints; for instance, CornerNet [41] detects objects by predicting pairs of top-left and bottom-right corners, while ExtremeNet [42] utilizes a combination of four extreme points and one center point. Conversely, center-focused methods shift attention to internal regions. CenterNet [22] treats the bounding box center as a positive sample to regress size offsets, Foveabox [43] jointly predicts semantic maps and coordinate mappings, and TTFNet [44] leverages Gaussian kernels to efficiently enrich training sample information around the center.

Unlike anchor-free detectors using uniform spatial grids without semantic grouping, STG introduces a lattice anatomically aligned with pedestrian proportions to provide a structured prior. Furthermore, while CenterNet-style detectors rely on isolated single-point predictions that fail when center features are corrupted, STG breaks this limitation through dual-layer Transformer interactions: I2GI aggregates inner-instance feature cues across body parts via axial cross-attention, while AIFI propagates inter-instance structural context from visible neighbors.

## 3. The Proposed Method

**Figure 2:**
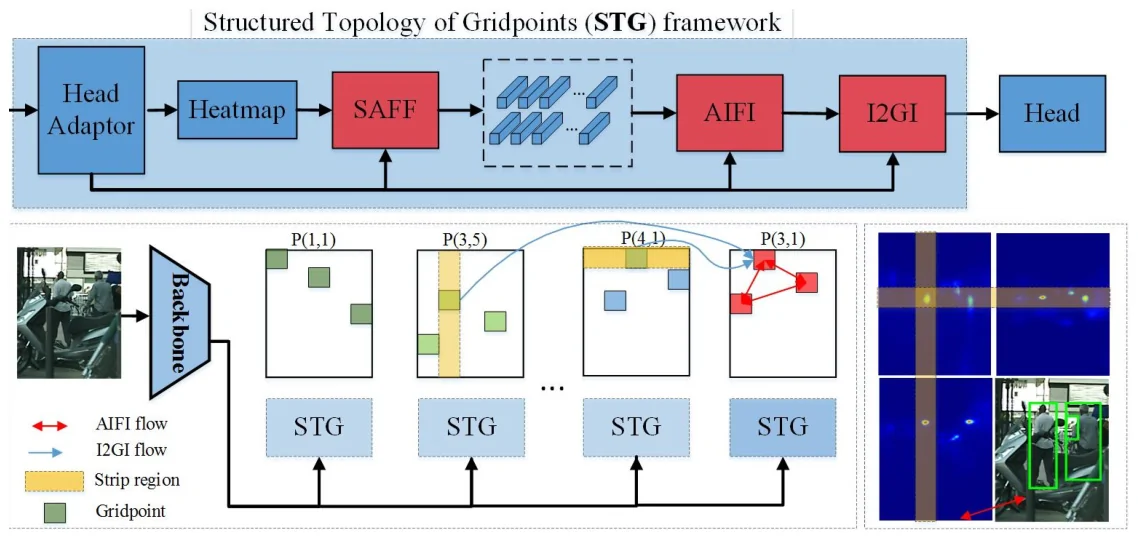
Overview of the STG framework. SAFF denotes salience-aware feature filtering module, AIFI denotes query-guided across-instance feature interaction, and I2GI denotes prior-guided inner-instance gridpoints interaction.

The pipeline of the proposed STG framework is shown in Figure 2. Our method consists of three main stages: SAFF, AIFI, and I2GI modules. The feature extraction module is based on the DLA34 backbone network; the multi-branch detection head module is similar to CenterNet [22] and adds other heatmap branches in parallel.

### 3.1. Definition of Gridpoints

**Figure 3:**
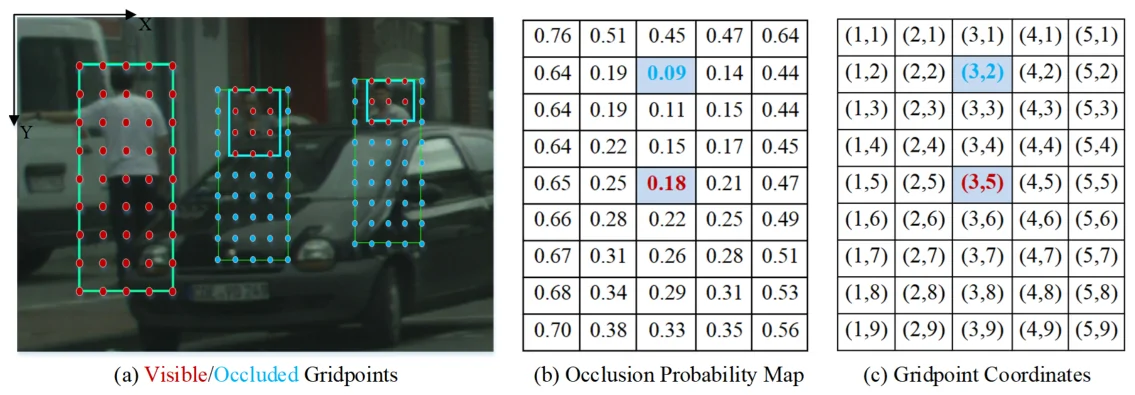
The definition of the gridpoints with coordinate (x,y). (**a**) The visible and occluded gridpoints are shown as red and blue points in green boxes. The horizontal and vertical directions represent the *x*-axis and *y*-axis. (**b**) The occlusion probability map (OPM) is calculated for each point on the CityPersons dataset. The gridpoints with lowest OPM (0.09) are located in position (3,2), while the OPM of center gridpoint (3,5) is 0.18. (**c**) The coordinates of each gridpoints.

To extract fine-grained, part-level visual evidence without relying on expensive visibility annotations, we formalize an endogenous geometric constraint array by segmenting the object proposal into a discrete lattice of gridpoints. As shown in Figure 3a, given a bounding box $B=\left[x_{c},y_{c},w,h\right]\in R^{4}$, where $\left(x_{c},y_{c}\right)$ represents the center coordinates, and w,h denote the width and height, respectively, we define a localized coordinate system within B, and project a discrete lattice topology G of size $w_{g}\times h_{g}$ (e.g., 5×9) onto the box interior, which closely matches the typical 1:2 aspect ratio of pedestrians. The normalized spatial coordinate of each individual gridpoint $P_{i,j}$ is mathematically derived via equidistant grid division (Equation (1)):

(1) $$P_{i,j}=\left(x_{c}-\frac{w}{2}+\frac{i-0.5}{w_{g}}\cdot w,\;y_{c}-\frac{h}{2}+\frac{j-0.5}{h_{g}}\cdot h\right),\;P=\left\{P_{i,j}\right\},$$

where $i\in \{1,\ldots ,w_{g}\}$ and $j\in \{1,\ldots ,h_{g}\}$ index the horizontal and vertical topological positions, respectively. *P* is a set of all $N_{g}=w_{g}*h_{g}$ gridpoints. Through this structured formulation, each $P_{i,j}$ natively anchors to a specific, intrinsic anatomical region of the pedestrian (e.g., $P_{3,2}$ aligns with the head region, while $P_{3,5}$ identifies the central torso), establishing a label-free, geometrically aligned feature localization mechanism.

Figure 3b illustrates the occlusion probability map of size 5×9, which is calculated on the CityPersons dataset. More specifically, for each gridpoint in the bounding box of training samples ($N_{total}=$19,654 pedestrians), the number of visible and occluded gridpoints is counted as $N_{i,j}^{v}$ and $N_{i,j}^{o}$; then, the probability of occlusion is counted for gridpoint $P_{i,j}^{o}=N_{i,j}^{o}/N_{total}$. For example, $P_{3,2}^{o}=0.09$ means only 9% of the gridpoint $P_{3,2}$ in the total training samples are occluded. As shown in Figure 3a, gridpoints in red denote visibility in the current bounding box, whereas gridpoints in blue represent occlusion occurring in the bounding box. In the original framework of CenterNet [22], only the primary center point $P_{3,5}$ is utilized for model training, but each gridpoint $P_{i,j}$ can be generalized as auxiliary in thegridpoint detection model with the same setting.

It is worth noting that our 5×9 multi-grid topology provides a relative semantic prior over the bounding box rather than rigid body-part keypoints. Driven by axial cross-attention in AIFI and I2GI, the feature propagation dynamically adapts to spatial variations. This design prevents performance degradation when encountering radical pose changes or arbitrary camera viewpoints, as evidenced by our strong generalization on the CrowdHuman and WiderPerson benchmark.

### 3.2. Problem Formulation

Given an input image $I\in R^{W\times H\times 3}$, the goal is to obtain *N* detection $D_{bbox}$ by the function $F_{backbone}$, $F_{neck}$, and $F_{head}$. The function $F_{backbone}$ acts as deep feature extraction from the raw RGB image *I* with a predefined backbone and output feature map of different scales. The $F_{neck}$ function is responsible for fusing the feature maps of disparate scales in order to obtain the feature $X\in R^{W^{\prime }\times H^{\prime }\times 64}$. The head function $F_{head}$ outputs a final detection of $D_{bbox}\in R^{N\times 5}$, with ψ(P,k) and feature map *X*, in which only *N* top scoring bounding boxes are selected. The feature extraction and detection head function is formulated in Equations (2) and (3), respectively.

(2) $$\begin{array}{c} X=F_{neck}\left(F_{backbone}\left(I\right)\right) \end{array}$$

(3) $$\begin{array}{c} D_{bbox}^{k}=\Phi \left(F_{head}^{k}\left(X,\psi \left(P,k\right)\right)\right),\;k\in \left\{1,2,\ldots ,N_{g}\right\} \end{array}$$

(4) $$\begin{array}{c} P_{i,j}=\psi \left(P,k\right),\;i=\left(\left(k-1\right)\;\mathrm{mod}\;w_{g}\right)+1,\;j=\left\lfloor \left(k-1\right)/w_{g}\right\rfloor +1 \end{array},$$

where $W^{\prime }=floor\left(W/s\right)$, $H^{\prime }=floor\left(H/s\right)$ are the width and height of feature map *X*, *W* and *H* are the width and height of input image *I*, and Φ represents the decoding function, which decodes the $F_{head}$ output into a bounding box representation. The feature map *X* produced by the backbone usually downsamples with 1/s of the original image size and s=4. ψ(P,k) selects $P_{i,j}$ from *P* for the *k*-th detection head to yield predictions. $F_{head}^{k}$ denotes detection heads from the $k^{th}$ gridpoint in STG, which are parallel in baseline.

Each gridpoint of STG within the bounding box can be employed as a regression task, offering distinct advantages when dealing with overlapping pedestrians. Each detection head $F_{head}^{k}$ is comprised of three distinct functions: $f_{hm}^{k}$, $f_{offset}^{k}$, $f_{size}^{k}$. The first function $f_{hm}^{k}$ learns to output a heatmap $D_{hm}^{k}$ in which the predefined gridpoint located have a high response. In order to locate the bounding box more accurately, it is also necessary to learn the offset part gridpoints for each gridpoint by the second function $f_{offset}^{k}$. Additionally, the width and height of the bounding box, which correspond to the gridpoint, are estimated by the third function $f_{size}^{k}$. They are formulated in Equation (5).

(5) $$\begin{array}{c} D_{hm}^{k}=f_{hm}^{k}\left(X\right),\;D_{offset}^{k}=f_{offset}^{k}\left(X\right),\;D_{size}^{k}=f_{size}^{k}\left(X\right) \end{array}$$

Finally, the decoding function Φ is mapped from the above result to $D_{bbox}^{k}$.

### 3.3. Saliency-Aware Feature Filtering (SAFF) Module

In crowded scenes, conventional dense cross-attention matching across all spatial locations introduces prohibitive computational overhead and non-target semantic clutter. To mitigate this, we introduce the SAFF module prior to global relation learning. It acts as a spatial feature selector, transforming the dense continuous feature manifold into a lightweight, discrete latent subspace focused strictly on highly salient foreground seeds.

Let $X\in R^{H^{\prime }\times W^{\prime }\times C}$ denote the dense feature map generated by the backbone network. To harvest the structural and geometry priors of pedestrian entities without relying on external visibility annotations, we employ a bank of decoupled, unshared prediction heads. We project *X* into an explicit spatial probability field $P\in R^{H^{\prime }\times W^{\prime }\times K}$, which estimates the geographic layout and structural saliency of the intrinsic gridpoint topology (e.g., K=2 for center P(3,5) and head P(3,2) gridpoints). Formally, the probability field at a specific spatial coordinate (x,y) is defined as follows:

(6) $$\begin{array}{c} P_{k}\left(x,y\right)=\sigma \left(W_{k}*X\left(x,y\right)+b_{k}\right),\;\forall k\in \left\{1,2,\ldots ,K\right\} \end{array},$$

where ∗ denotes the 2D spatial convolution, $W_{k}$ and $b_{k}$ represent the learnable parameters of the *k*-th prediction branch, and σ(·) is the element-wise Sigmoid function constraining the saliency intensity to (0,1). To prune background noise and sparsify the continuous representation space, we apply a dynamic confidence gating function on $P_{k}$ to filter out non-object layout areas. By validating spatial coordinates whose peak structural responses exceed a critical threshold τ, we distill the high-confidence target anchor set Ω:

(7) $$\Omega =\left\{\left(x_{i},y_{i}\right)\;|\;\max P_{k}\left(x,y\right)>\tau \right\},\;\mathrm{with}\;\left|\Omega \right|=M\ll \left(H\times W\right),$$

where *M* represents the total number of high-confidence sparse foreground candidates selected for subsequent cross-instance reasoning. Guided by this discrete set Ω, the original dense feature tensor is compactly reconstructed into a downscaled sparse feature matrix $X_{sparse}\in R^{M\times C}$:

(8) $$X_{sparse}={\left[X\left(x_{1},y_{1}\right),X\left(x_{2},y_{2}\right),\ldots ,X\left(x_{M},y_{M}\right)\right]}^{⊤}.$$

By utilizing $X_{sparse}$ as the sparse keys and values in the subsequent AIFI layer, the computational complexity is successfully bounded from $O(N\cdot H^{2}\cdot W^{2}\cdot C)$ down to $O(N\cdot M^{2}\cdot C)$, effectively shielding the relation learning from feature cross-talk and background clutter.

### 3.4. Gridpoints Feature Interaction

As illustrated in Figure 2, the proposed method is a gridpoint feature interaction network, which is capable of facilitating feature interaction across two dimensions: global foreground features and individual instance features. This method comprises two components: AIFI and I2GI modules, which will be described in the following.

For a more clear explanation, we utilize two gridpoints $P_{i,j}$, $P_{l,m}$, for gridpoint feature interaction. Given an input feature map *X* from the backbone $F_{backbone}$, salient feature filtering is first employed with anchor set Ω=SAFF(X); across-instance feature interaction of gridpoints $P_{i,j}$, $P_{l,m}$ are used for interaction $X_{i,j}=AIFI_{i,j}\left(X,\Omega \right)$, $X_{l,m}=AIFI_{l,m}\left(X,\Omega \right)$ separately. Then, inner-instance gridpoint interaction is employed for different positions from each instance, ${\hat{X}}_{i,j}$, ${\hat{X}}_{l,m}$ = $I2GI(X_{i,j},X_{l,m}$). Finally, each enhanced feature map ${\hat{X}}_{i,j}$ or ${\hat{X}}_{l,m}$ can be used for the final output feature map, which serves as the input to the detection head $F_{head}$.

#### 3.4.1. Across-Instance Feature Interaction (AIFI) Module

**Figure 4:**
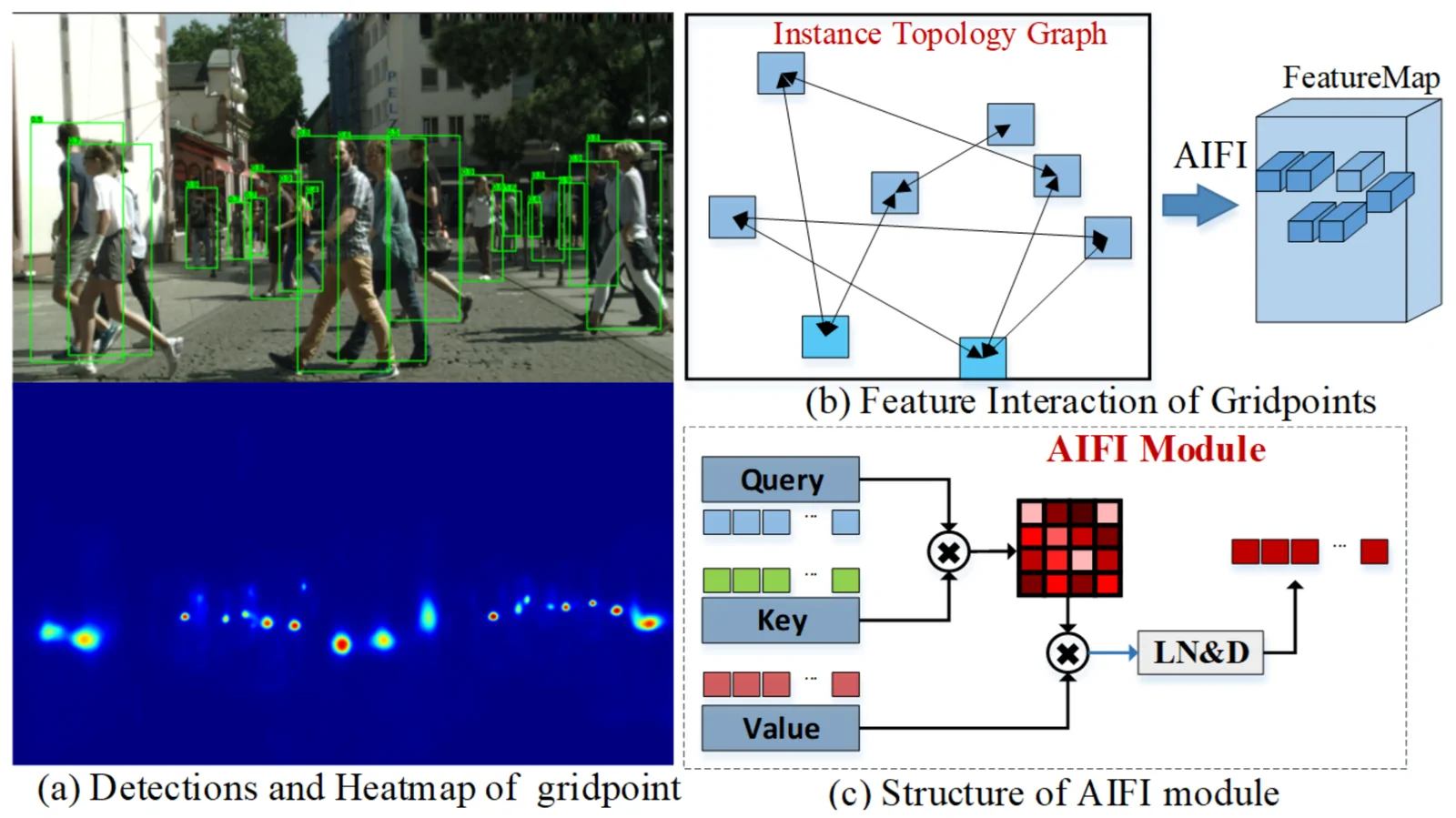
Across-Instance gridpoint interaction with AIFI modules: (**a**) presents detection results and the heatmap of the center branch, while (**b**,**c**) illustrate the gridpoint interactions and the AIFI module, respectively. Green blocks represent raw gridpoint feature channels, while orange and purple blocks denote transformed Query (*Q*), Key (*K*), and Value (*V*) representations. Solid directional lines trace self-attention paths.

In this section, we introduce the AIFI Module (Figure 4c), which is formulated as a topological sparse self-attention mechanism. The insight behind AIFI is to reformulate the fragmented candidate instances into a dynamic, non-local gridpoint topology graph (Figure 4b). In this graph, each active object candidate serves as a node, allowing highly visible pedestrians to act as topological anchors that propagate contextual layout constraints to their heavily obscured neighbors, thereby reconstructing the missing holistic semantics. The full procedure of AIFI module comprises three main steps: Sparse Gridpoints Projection, Topological Relational Reasoning, and Adaptive Residual Connection.

**(1) Sparse Gridpoints Projection**: Rather than executing unconstrained global attention across the entire dense continuous feature map *X*, AIFI leverages the explicit spatial probability field generated by the preceding SAFF module. By identifying the Top-K peak responses on the gridpoint saliency heatmaps (Figure 4a), we extract the coordinates of the most optimal candidate target seeds. Concurrently, the dense feature vectors at these discrete topological locations are collected to construct the sparse feature matrix $X_{sparse}\in R^{M\times C}$. Through this projection, we purge non-target semantic clutter and background noise beforehand, linearizing the candidate flow into a highly purified foreground item set.

**(2) Topological Relational Reasoning**: To capture the macro-structural context of the crowd and propagate relational constraints among distinct pedestrians, we orchestrate a scaled dot-product self-attention mechanism over the sparse matrix $X_{sparse}$. The query, key, and value matrices ($Q_{A},K_{A},V_{A}\in R^{M\times C}$) are projectively derived from the same $X_{sparse}$, which is formulated in Equation (9):

(9) $$\begin{array}{c} Q_{A}=X_{sparse}W_{Q}^{A},\;K_{A}=X_{sparse}W_{K}^{A},\;V_{A}=X_{sparse}W_{V}^{A} \end{array},$$

where $W_{Q}^{A},W_{K}^{A},W_{V}^{A}\in R^{C\times C}$ denote the learnable parameter weights that project the tokens into a shared relational embedding space. The global crowd interaction and affinity propagation are subsequently defined in Equation (10):

(10) $$\begin{array}{c} X_{AIFI}=\mathrm{Softmax}\left(\frac{Q_{A}K_{A}^{⊤}}{\sqrt{C}}\right)V_{A} \end{array}.$$

Through this sparse self-attention graph, candidate nodes that are highly visible and retain complete semantic structures act as topological anchors, spontaneously transferring missing contextual layouts to heavily obscured neighbor nodes.

**(3) Adaptive Residual Connection**: To complete the collaborative reasoning loop and ensure uninterrupted gradient flow during end-to-end training, the relation-enhanced sparse features $X_{sparse}$ are mapped back to their original spatial coordinates. We define this backward integration as an adaptive residual connection process, which is defined in Equation (11):

(11) $$\begin{array}{c} X_{AIFI}=X+S_{\mathrm{index}}\left(X_{AIFI}\right) \end{array},$$

where $S_{\mathrm{index}}\left(\cdot \right)$ represents an exact indexical scatter operator that maps the discrete *K* tokens back into the 3D dense tensor coordinates. Consequently, while the non-object background feature regions are safely shielded from degradation, the critical foreground pedestrian features are dynamically updated with rich, crowd-level global constraints. This design gracefully transforms mutual occlusion from a disruptive hazard into a predictive structural clue, optimizing the unified feature volume for robust boundary regression.

#### 3.4.2. Inner-Instance Gridpoints Interaction (I2GI) Module

**Figure 5:**
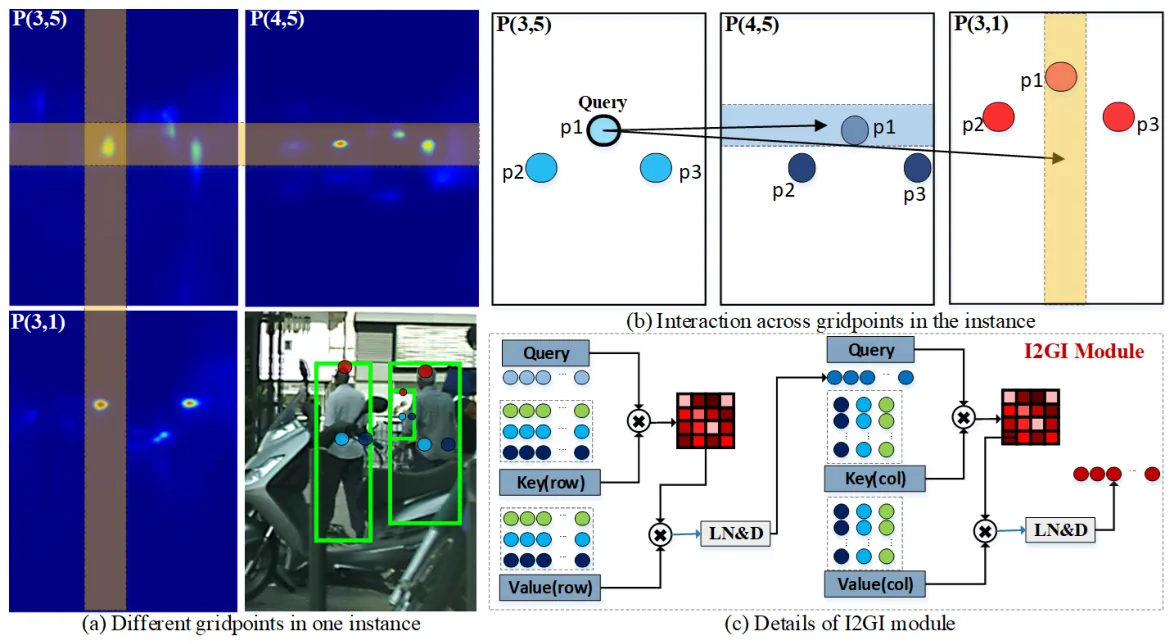
Inner-instance gridpoint interaction with I2GI module: (**a**) illustrates the gridpoint and corresponding horizontal and vertical neighbors; (**b**) shows the interaction with three gridpoints of object p1; and (**c**) shows details of I2GI module. Thermal color scales denote feature response intensity, ranging from blue (low activation) to bright red (high activation).

While the AIFI module captures macroscopic crowd constraints, rectifying localized, part-level feature corruption within an individual pedestrian requires a finer, structurally aware lens. To mitigate intra-class semantic erosion caused by directional blockages, we introduce the I2GI Module (Figure 5). Instead of utilizing unconstrained attention over the entire object space, I2GI introduces a bidirectional local axial cross-attention mechanism that propagates semantic context strictly along the horizontal and vertical topological axes of the intrinsic gridpoint lattice.

**(1) Local Grid-Neighborhood Formulation**: Let $X_{AIFI}\in R^{H\times W\times C}$ denote the dense feature map enhanced by the preceding global relation learning stage. To systematically guide feature alignment without external visibility annotations, we define a localized geometric neighbor set $N(P_{i,j})$ for each gridpoint candidate $P_{i,j}$. This set is explicitly decoupled into horizontal and vertical structural adjacency paths, which is formulated in Equation (12):

(12) $$\begin{array}{c} N\left(P_{i,j}\right)=\left\{P_{i,\cdot }\;|\;\mathrm{Horizontal}\;\mathrm{Axis}\right\}\cup \left\{P_{\cdot ,j}\;|\;\mathrm{Vertical}\;\mathrm{Axis}\right\} \end{array},$$

where $P_{i,\cdot }$ represents the set of all gridpoints sharing the same row, and $P_{\cdot ,j}$ collects all gridpoints sharing the same column within the instance lattice. This alignment directly models the directional nature of real-world occlusions.

**(2) Bidirectional Axial Cross-Attention**: The micro-level semantic completion is executed by establishing a query-key relational loop bounded within the neighborhood $N(P_{i,j})$. For a specific gridpoint target $P_{i,j}$, its query vector $q_{i,j}\in R^{1\times C}$ is extracted from its own localized feature representation. Concurrently, the key and value matrices $K_{i,j}^{\mathrm{axial}}$ and $V_{i,j}^{\mathrm{axial}}\in R^{(W_{g}+H_{g}-1)\times C}$ (Equation (13)) are dynamically aggregated from the feature vectors of its horizontally and vertically adjacent neighbors in $N(P_{i,j})$:

(13) $$\begin{array}{c} K_{i,j}^{\mathrm{axial}}=\mathrm{Concat}\left(X_{AIFI}\left(P_{i,\cdot }\right),X_{AIFI}\left(P_{\cdot ,j}\right)\right)\cdot W_{K}^{I}\\ V_{i,j}^{\mathrm{axial}}=\mathrm{Concat}\left(X_{AIFI}\left(P_{i,\cdot }\right),X_{AIFI}\left(P_{\cdot ,j}\right)\right)\cdot W_{V}^{I} \end{array},$$

where $W_{K}^{I}$ and $W_{V}^{I}\in R^{C\times C}$ are learnable projection weights. The localized semantic compensation is subsequently formulated via a prior-guided axial cross-attention paradigm (Equation (14)):

(14) $$\begin{array}{c} I2GI\left(q_{i,j}\right)=\mathrm{Softmax}\left(\frac{q_{i,j}{\left(K_{i,j}^{\mathrm{axial}}\right)}^{⊤}}{\sqrt{C}}\right)V_{i,j}^{\mathrm{axial}} \end{array}.$$

**(3) Part-Level Semantic Completion**: Through this axial cross-attention scheme, the network effectively constructs an intra-instance part feature propagation mechanism. When a pedestrian’s lower body is heavily obscured horizontally (e.g., by a vehicle barrier) or a side profile is blocked vertically (e.g., by a vertical pole), the features at the heavily corrupted gridpoints function as weak queries. The attention distribution automatically suppresses the corrupted inputs within $K_{i,j}^{\mathrm{axial}}$ and steers the weight gains toward unoccluded, high-confidence vertical or horizontal neighbor nodes that retain clear visual evidence. These reliable adjacent features seamlessly broadcast their intact semantic structures along the intersecting grid axes, acting as localized evidence chains to restore the degraded object representations. By aggregating these directional cues, the I2GI module eliminates intra-class feature confusion and outputs an occlusion-gated, highly discriminative representation optimized for precise bounding box regression.

#### 3.4.3. Unified Propagation Pipeline

To explicitly unify the feature update chain, the complete data flow from backbone feature maps $X_{in}$ to the detection head input $X_{head}$ is formulated as follows:

(15) $$X_{head}=M_{I2GI}\left(M_{AIFI}\left(M_{SAFF}\left(X_{in}\right)\right)\right),$$

where $M_{SAFF}$, $M_{AIFI}$, and $M_{I2GI}$ represent the sequential transformations of our three modules:

- **SAFF** extracts sparse aligned gridpoint features: $X_{SAFF}=M_{SAFF}\left(F_{in}\right)$.
- **AIFI** propagates across-instance spatial context: $X_{AIFI}=M_{AIFI}\left(X_{SAFF}\right)+X_{SAFF}$.
- **I2GI** applies axial topology completion to yield the head input: $F_{head}=M_{I2GI}\left(X_{AIFI}\right)$.

It is worth mentioning that I2GI operates within an end-to-end framework where gridpoints are predicted via dense heatmaps, and their association with instance-specific topologies is established through explicit spatial-index mapping.

### 3.5. Loss Function

#### 3.5.1. Detection Head of Single Gridpoint

The single gridpoint detection module contains three main outputs, the gridpoint degree (confidence level), the object size, and the coordinate offset. Similar to CenterNet, the model training process in this paper uses a similar gridpoint loss function, object size loss function, and gridpoint offset loss function, each of which is described in detail below.

**Pointness Loss:** The probability prediction of the location of the gridpoint k uses Focal Loss, as shown in Equation (16).

(16) $$L_{pt}^{k}=\left\{\begin{array}{c} \frac{-1}{N}\sum_{x,y}{\left(1-{\hat{Y}}_{x,y}^{k}\right)}^{\alpha }log\left({\hat{Y}}_{x,y}^{k}\right),if\;Y_{x,y}^{k}=1\\ \frac{-1}{N}\sum_{x,y}{\left(1-Y_{x,y}^{k}\right)}^{\beta }{\left({\hat{Y}}_{x,y}^{k}\right)}^{\alpha }log\left(1-{\hat{Y}}_{x,y}^{k}\right),others \end{array}\right.,$$

where α and β are hyperparameters of Focal Loss, set α=2 and β=4 in this paper, which are standard values directly adopted from original CenterNet configurations. *N* is the number of candidate target gridpoints in image *I*, $Y_{x,y}^{k}$ represents the probability that the current location (x,y) is a pedestrian gridpoint, and $Y_{x,y}^{k}=1$ represents the true value of the current location being a gridpoint.

**Width and Height Loss:** The width–height loss function corresponding to the gridpoint k uses the L1 loss, as shown in Equation (17).

(17) $$\begin{array}{c} L_{size}^{k}=\frac{1}{N}\sum_{j=1}^{N}\left|{\hat{S}}_{pj}^{k}-S_{j}^{k}\right| \end{array},$$

where ${\hat{S}}_{pj}^{k}$ is the predicted size of the *j*th target using gridpoint *k* and $S_{j}^{k}$ is the true value of that target size.

**Offset Loss:** The offset loss function corresponding to the gridpoint k also uses the L1 loss, as shown in Equation (18).

(18) $$\begin{array}{c} L_{off}^{k}=\frac{1}{N}\sum_{p}\left|{\hat{O}}_{\hat{p}}^{k}-\left(\frac{p}{R}-{\hat{P}}^{k}\right)\right| \end{array},$$

where *p* indicates the presence of a critical point at the current location; therefore, this loss does not need to be calculated for locations that are not critical. In summary, the loss function $L^{k}$ for the whole network is shown in Equation (19).

(19) $$\begin{array}{c} L_{det}^{k}=L_{pt}^{k}+\lambda_{size}*L_{size}^{k}+\lambda_{off}*L_{off}^{k} \end{array}.$$

In this paper, $\lambda_{size}$ is set to 0.1 and $\lambda_{off}$ is set to 1. The entire network is trained by minimizing this loss function for predicting the heat map ${\hat{Y}}^{k}$, the offset ${\hat{O}}^{k}$, and with size ${\hat{S}}^{k}$ for all gridpoints in the image.

#### 3.5.2. Detection Head of All Gridpoints

To enable the multi-branch detection module proposed in this paper, which contains K gridpoint detection branches (e.g., K = 2 for head and center gridpoints), the corresponding output features and regression targets need to be learned independently through multiple tasks. The total loss functions for branches 1,2,…,K are obtained as $L^{1}$, $L^{2}$, …, $L^{k}$, according to Equation (19). Therefore, the loss function of multiple gridpoint-based pedestrian detection is shown in Equation (20).

(20) $$\begin{array}{c} L_{det}^{K}=\sum_{k=1}^{K}L_{det}^{k} \end{array}.$$

## 4. Experiments

### 4.1. Datasets

**CityPersons Dataset [45]:** The CityPersons dataset is recorded across 18 different cities in Germany with 3 different seasons and various weather conditions. The dataset includes 5000 images (2975 for training, 500 for validation, and 1525 for testing) with 35,000 manually annotated persons, plus 13,000 ignored annotations.

**CrowdHuman Dataset [46]:** The images are crawled from Google image with 25,000 images in total. There are 15,000, 4370, and 5000 images for training, validation, and testing, respectively. There are 339,565 person annotations in total, with both high diversity (339,565 unique persons) and density (22.64 persons per image in average).

**WiderPerson Dataset [47]:** WiderPerson is a dense human detection dataset under multiple different scenes and is not limited to traffic scenes. It contains 8000, 1000, and 4382 images for training, validation, and testing sets, respectively. We use exactly the same division as the original dataset. It contains five types of annotations: pedestrians, riders, partially visible persons, crowds, and ignored regions.

### 4.2. Implementation Details

Our method is implemented using the PyTorch 1.8.1 framework on a Ubuntu 18.04 server with CPU i7-9700, 64G Memory, and Nvidia RTX 3080 Ti GPU. The training phase takes 50 epochs, with a batch size of 2. The SGD optimizer is used with the initial learning rate of $1.0\times 10^{-2}$ and the momentum of 0.937. In addition, the weight decay factor is 0.0005 and the learning rate decay method is cosine annealing. For the CityPersons dataset, the input size of images is 1024 × 2048 for training and 1024×2048 for testing. In addition, mosaic and random flipping are used for data augmentation. For the CrowdHuman and WiderPerson datasets, the input image sizes are set to 800×1216 and 768×1152, respectively, while all other training configurations remain consistent with those used for CityPersons. The filtering threshold in SAFF is set to 0.45, and the number of candidate points chosen is M=50.

**Evaluation Metric:** The log miss rate averaged over the false positive per image (FPPI) range of [$10^{-2}$, 100] (MR) is used (lower is better). CityPersons dataset is split into different subsets according to the occlusion ratio and height of pedestrians. Following [45], we mainly report results on Reasonable (R) and Heavy Occlusion (HO) setting. The occlusion ratio in R is smaller than 35% and the occlusion ratio in HO is between 35% and 80%. Only pedestrians with height over 50 pixels are considered in the subsets. In addition, we also explore the results of our model under the reasonable small scale subset (RS) of the CityPersons dataset and the whole dataset (A), the RS subset with height in [50, 75] and visibility in [0.65, 1].

### 4.3. Ablation Study

In this section, we perform ablation experiments and verify the effectiveness of the proposed method. In the following, the CenterNet network based on DLA34 is used as the baseline method.

#### 4.3.1. Effects of Different Gridpoints’ Position

We perform the experiment according to the definition of the gridpoints in Figure 3, the upper left gridpoint of each bbox denotes $P_{1,1}$, and the lower right gridpoint denotes $P_{5,9}$. We show the performance of different R and HO subsets of gridpoints in Table 1. As shown in Table 1, when the gridpoint is in the upper half of the bbox, HO generally has better performance, peaking at position $P_{3,2}$. However, when the gridpoint is in the middle of the bbox, the performance of the R subset is generally better than either the left or right, peaking at position $P_{3,5}$. This shows that the gridpoints in the upper half usually have a better effect on occlusion, and the gridpoints in the middle have a better versatility than the points on either side.

In addition, we note that in Figure 3b, the occlusion probability of the gridpoints located inside the bbox shows a steep decline compared to the gridpoints located at the boundary, while the performance change in Table 1 is smooth. This may be due to the fact that the annotations of the visible bbox of the dataset usually do not contain the boundary points of the full bbox, and our statistical algorithm is responsible for counting whether the gridpoint at each position is in the visible bbox.

Statistically, $P_{3,2}$ and $P_{3,5}$ correspond to the head/upper torso and the body center, respectively, which represent regions with the highest semantic density and lowest occlusion variance across the dataset. Consequently, these two key points are selected as the optimal anchor gridpoints to effectively guide the feature aggregation in the subsequent SAFF and AIFI modules.

Another interesting phenomenon emerges when examining the gridpoint coordinates in Figure 3c: the points residing along the central column, specifically $P_{\left(3,:\right)}$, consistently achieve superior performance across all metrics. Furthermore, as gridpoints become closer to the vertical centerline, the *MR* on the R subset exhibits a steady decline. A similar trend is observed under HO, where $P_{\left(2,2\right)}$, located adjacent to the central point $P_{\left(3,2\right)}$, yields a noticeably lower miss rate than its more distant counterparts.

#### 4.3.2. Effects of SAFF and AIFI for Different Gridpoints

In the previous section, the experimental results show that the gridpoint $P_{3,5}$ and the gridpoint $P_{3,2}$ can represent two types of scenes: the general case and the occluded case. Therefore, we choose them as the baseline for the SAFF module and the AIFI module in this section. As shown in Table 2, integrating the SAFF and AIFI modules consistently suppresses the miss rate across all evaluation metrics, regardless of the gridpoint positions. Specifically, when deploying the modules at gridpoint $P_{3,2}$—which targets occluded cases—the miss rate for HO drops significantly from 34.40% to 31.67% (a 2.73% absolute improvement). This demonstrates that capturing cross-instance correlations via AIFI effectively mitigates feature ambiguity in crowded scenes. Concurrently, utilizing gridpoint $P_{3,5}$ yields the upper-bound performance for general cases, where the metrics for R, RS, and A are reduced to their minimums at 8.14%, 11.44%, and 29.73%, respectively. These results can verify that the SAFF and AIFI modules successfully enhance the backbone features and boost the model’s robustness under diverse environmental complexities.

#### 4.3.3. Influence of Auxiliary Gridpoints Selection

To evaluate the impact of the auxiliary gridpoint selection within the I2GI module, we fix the primary gridpoint at (3,5) and systematically vary the paired auxiliary gridpoints along the vertical axis, as detailed in Figure 3c.

As shown in Table 3, the empirical results across both CityPersons and WiderPerson datasets consistently reveal that the relative spatial proximity of auxiliary gridpoints to the vertical centerline plays a crucial role in representation learning. Specifically, pairing the primary point (3,5) with the auxiliary point (3,2)—which resides nearest to the upper central region—achieves the best overall detection performance. On CityPersons, it yields the lowest log-average miss rates (${\mathrm{MR}}^{-2}$) of 7.41%, 10.46%, and 32.05% on the R, RS, and HO subsets, respectively. This superior performance transfers directly to the more crowded WiderPerson benchmark, where the (3,2) configuration attains both the lowest *MR* (40.74%) and the highest AP (90.22%). As the auxiliary point moves further away from the central region toward the outer boundaries (e.g., (3,6) or (3,7)), a consistent degradation in performance is observed across both datasets. For instance, on CityPersons, the *MR* on the Reasonable subset deteriorates to 9.23%, while on WiderPerson, the *MR* increases to 41.68% and the AP drops to 88.83%.

This trend aligns with our physical intuition: gridpoints located near the human body’s vertical centerline carry substantially higher semantic stability and lower susceptibility to background noise. Consequently, aggregating features from central auxiliary gridpoints provides richer, more discriminative complementary evidence for reconstructing degraded pedestrian representations across diverse real-world scenarios.

#### 4.3.4. Effect of Different Modules (SAFF, AIFI, and I2GI)

To quantify the individual and synergistic contributions of SAFF, AIFI, and I2GI, we conduct a component-wise ablation study on CityPersons and WiderPerson in Table 4. As observed, introducing SAFF alone enhances feature resolution for occluded and dense targets, reducing HO MR from 35.03% to 34.20%. Incorporating AIFI or I2GI individually further improves detection across both datasets, with I2GI alone driving MR down to 7.87% on CityPersons *R* and 41.03% on WiderPerson MR. When combining all three modules, our full network achieves the best overall performance (7.41% Reasonable MR on CityPersons and 90.22% AP on WiderPerson), demonstrating that spatial feature alignment (SAFF), across-instance context propagation (AIFI), and inner-body topological completion (I2GI) complement each other effectively to boost pedestrian detection under severe occlusion.

We have also conducted an independent ablation analysis on the Reasonable Small (RS) subset (Table 4). Solely integrating SAFF slightly degrades performance (12.42% vs. 11.90%MR) because unconstrained cross-scale fusion tends to amplify high-frequency background noise for low-resolution instances. However, when paired with AIFI and I2GI, which enforce strict spatial topology constraints, SAFF supplies essential high-frequency context without background corruption. Together, the full framework achieves a overall best miss rate of 10.46% on the RS subset, representing a 1.44% absolute improvement over the baseline.

#### 4.3.5. Gridpoint Settings and Attention Mechanism Ablation

To validate gridpoint selection, ordering, and attention mechanisms, we evaluate various single-gridpoint (SG) and multi-gridpoint (MG) configurations in Table 5. Multi-point aggregation (P(3,5)+P(3,2)) significantly outperforms single-point baselines (e.g., Reasonable *MR* drops from 9.61% to 8.78%), confirming the benefit of complementary spatial priors. Furthermore, setting P(3,5) as the primary reference consistently surpasses the reversed order (P(3,2)+P(3,5)), proving the central body region provides a more stable anchor than the head. Introducing Self-Attention (MG+SA) and Cross-Attention (MG+CA) progressively enhances feature alignment. Notably, expanding to 3-gridpoint setups (e.g., adding P(2,5) or P(4,5)) slightly degrades performance (*MR* rises to 8.26–8.48%), indicating that adding excess sampling points introduces feature redundancy and noise. Our Full STG model achieves the optimal performance across all metrics (7.41% *MR* on Reasonable and 32.05% on Heavy Occlusion), confirming the efficacy of our structured topological design over naive point addition.

#### 4.3.6. Comparisons with Different Backbones

In order to assess the efficacy and applicability of our proposed module, we conducted experiments on detectors utilizing two distinct backbones: Resnet18 and DLA34. As demonstrated in Table 6, the results on the CityPersons dataset reasonable subsets demonstrate that our approach outperforms the baseline method by 5.36% and 1.37% on ResNet18 and DLA34, respectively. Consequently, it can be concluded that the proposed module is applicable to different backbones and is effective under different evaluation metrics.

#### 4.3.7. Ablation of Threshold τ

To evaluate the impact of the foreground candidate threshold τ (Equation (7)) on detection performance, we conduct a sensitivity ablation study by varying τ from 0.30 to 0.55 in Table 7. As shown in Table 7, setting τ too low (0.30) leads to a higher miss rate (MR of 10.25%), as redundant and noisy background gridpoints are mistakenly included in the foreground candidate set Ω. Conversely, an overly strict threshold (τ=0.55) prematurely discards informative topological features, causing MR to degrade to 10.12%. The optimal detection performance (9.35%) *MR* is achieved at τ=0.45, striking a precise balance between proposal recall and feature purity.

### 4.4. Comparison with State-of-the-Arts Methods

**CityPersons dataset [45]:** The quantitative comparison results on the CityPersons validation set are presented in Table 8. Utilizing a standard DLA-34 backbone with enhanced feature extraction, our STG framework achieves SOTA performance across all evaluation subsets. Moreover, our model demonstrates substantial improvements on highly challenging subsets, securing a competitive 10.46% *MR* on RS and 32.05% on HO without relying on targeted small-object detection techniques.

**CrowdHuman dataset** [46]: The experimental comparisons on the CrowdHuman dataset are given in Table 9. To validate the generalization performance of the method proposed in this paper for occlusion, comparative experiments are conducted on the CrowdHuman dataset, evaluating against the official standard performance metrics. These results demonstrate the robustness and generalization ability of the proposed method, which performs better in pedestrian detection tasks in complex scenes, achieving the lowest miss rate of 44.2%. While our STG achieves the lowest *MR* (44.2%), its AP (85.2%) is slightly lower than some holistic detectors like Sparse R-CNN and DiffusionDet. This accuracy–recall trade-off arises because STG prioritizes fine-grained part-level feature completion to minimize miss rates under severe occlusions, which can amplify ambiguous background noise and elevate false positives.

**WiderPerson dataset** [47]: Table 10 shows the comparison of our method with existing methods on the WiderPerson dataset. We refer to [47] for results obtained on a hard subset of annotations, which contain all the boxes larger than 20 pixels in height. To verify the generalization performance of the method proposed in this paper for camera viewpoint variations, our STG detector was experimentally compared with the latest state-of-the-art methods on the WiderPerson dataset. The official standard performance metrics of recall, average accuracy AP, and mean miss rate *MR* were evaluated. The recall, AP, and *MR* of the algorithm presented in this paper on the WiderPerson dataset is 93.51%, 90.22%, and 40.74%, respectively. The proposed method demonstrates slightly better performance in terms of both Recall and AP evaluation metrics, when compared to IterDet 1iter. Conversely, it exhibits competitive performance in *MR* and AP evaluation metrics, when compared to IterDet 2iter, which can be regarded as a compromisation method.

### 4.5. Detection Speed Comparisons

Table 11 evaluates the runtime efficiency and computational complexity of our proposed framework against representative detectors under the same hardware environment. At a high image resolution of 1024×2048, our method achieves an average processing speed of 16 FPS, with 28.52 M parameters and 568.52 G FLOPs.

Compared with traditional detectors like FCOS and Faster R-CNN, our model not only operates at a higher inference speed (16 FPS vs. 15 FPS) but also substantially reduces computational burden—saving over 28% in FLOPs and reducing parameters by up to 31%. Although YOLOv5m exhibits higher throughput due to its lightweight design, our approach achieves significantly superior detection accuracy under severe occlusion and high density. These results demonstrate that our framework strikes a balance between accuracy, parameter efficiency, and execution speed, making it viable for real-world surveillance deployments.

#### Quantitative Analysis of FP and FN

To assess the error modes, we report the total False Positives (FP) and False Negatives (FN) across the 4370 validation images in Table 12. Under a fixed threshold of 0.35, our STG achieves the lowest missed detections (21,560 total FN, 44.20% MR), outperforming Sparse R-CNN (22,080 FN) and DiffusionDet (29,362 FN) under severe occlusion. While Sparse R-CNN and DiffusionDet yield higher overall AP with fewer false alarms (1834 and 1686 FP), STG at lower thresholds prioritizes recall for heavily occluded instances. Furthermore, raising STG’s threshold to 0.45 and 0.55 significantly reduces FP from 5452 down to 2490 and 950, respectively, demonstrating that STG’s false alarms can be effectively suppressed to match or exceed the precision levels of query-based models by tuning the confidence score cut-off.

### 4.6. Qualitative Analysis

**Figure 6:**
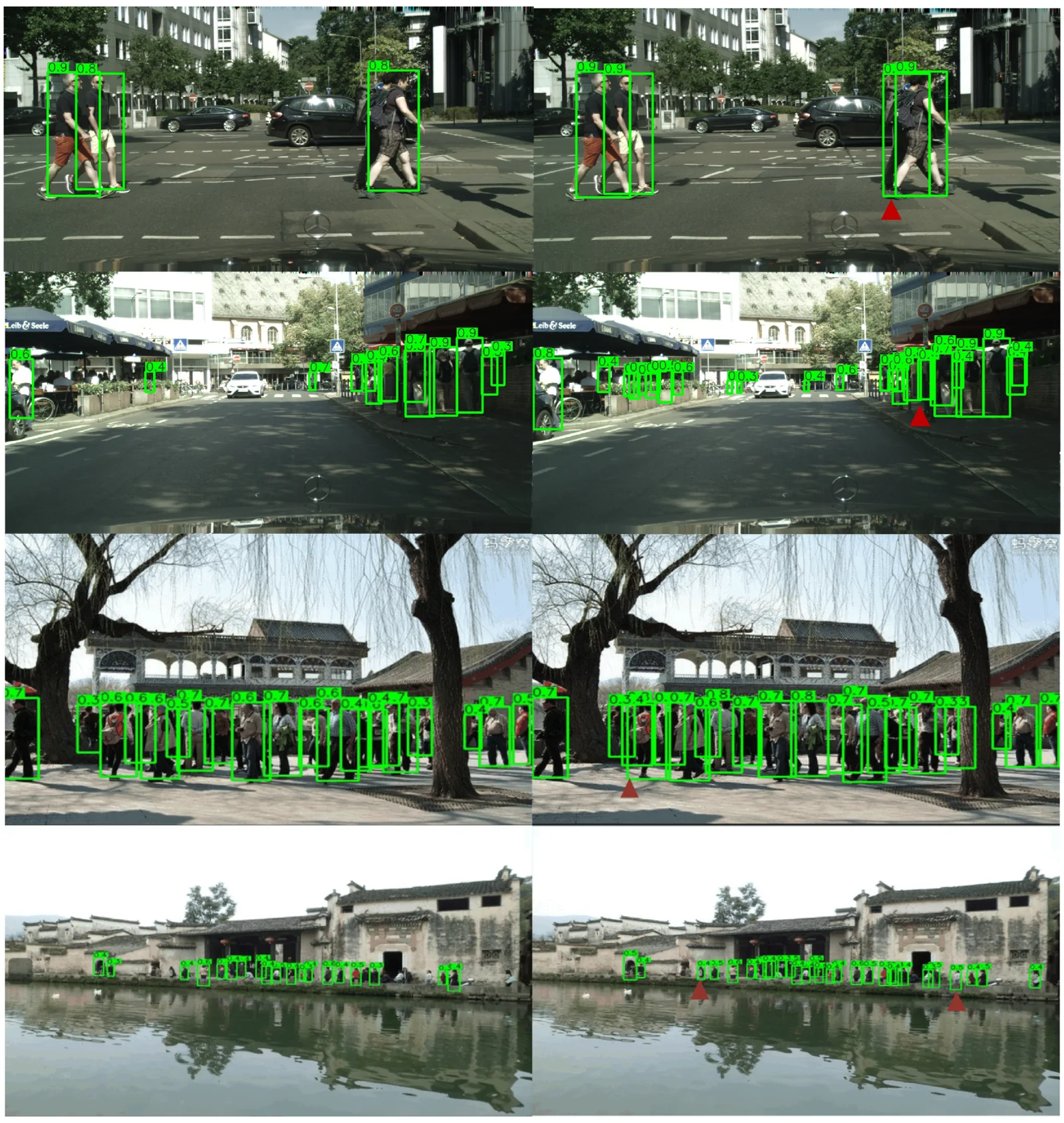
Visualization results of images on CityPersons and WiderPerson dataset. Left column is the baseline method and right column is our method. The red triangle flag represents the missed detection of the baseline method.

Figure 6 (top) presents the visualization results on the CityPersons dataset. As illustrated in the first column, the baseline approach encounters difficulties in detecting every pedestrian in a crowded environment. However, our method is capable of identifying and locating targets by aggregating the information in the feature map. Furthermore, the baseline approach results in a higher number of false positives. Nevertheless, our method is able to leverage global spatial location information and correlations between different objects to capture highly differentiated features. In order to facilitate a more comprehensive comparison, we have also chosen to visualize both our method and the baseline method on the WiderPerson dataset (Figure 6 (bottom)). It can be observed that there is a certain degree of improvement for small targets and occluded targets.

**Figure 7:**
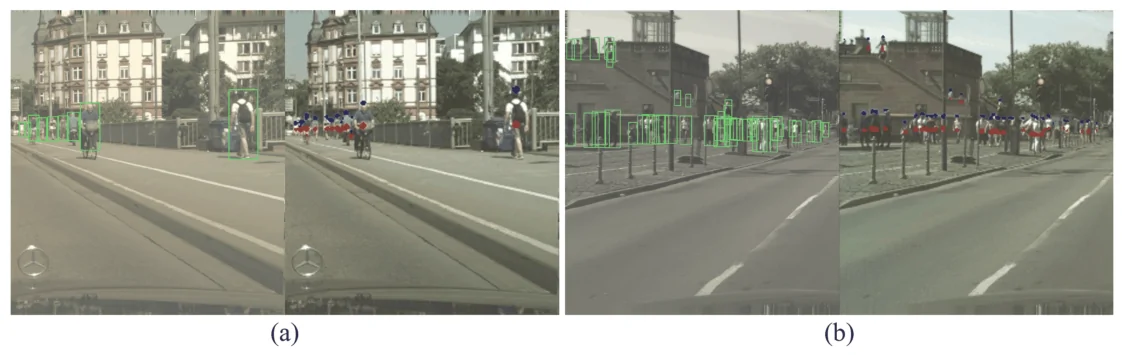
The visualization of gridpoints at different positions in scenario (**a**,**b**). In each scenario, the figure on the left represents Ground Truth, and the figure on the right is a visualization of the gridpoint in the downsampled image. Red points represent the center gridpoints, while blue points represent the head gridpoints.

Furthermore, as illustrated in Figure 7, we present the locations of the gridpoints that have been adopted differently in the CityPersons dataset. We utilize point features at these locations for cross-attention, thereby enabling the model to model at long distances without incurring significant computational costs.

### 4.7. Limitation

**Figure 8:**
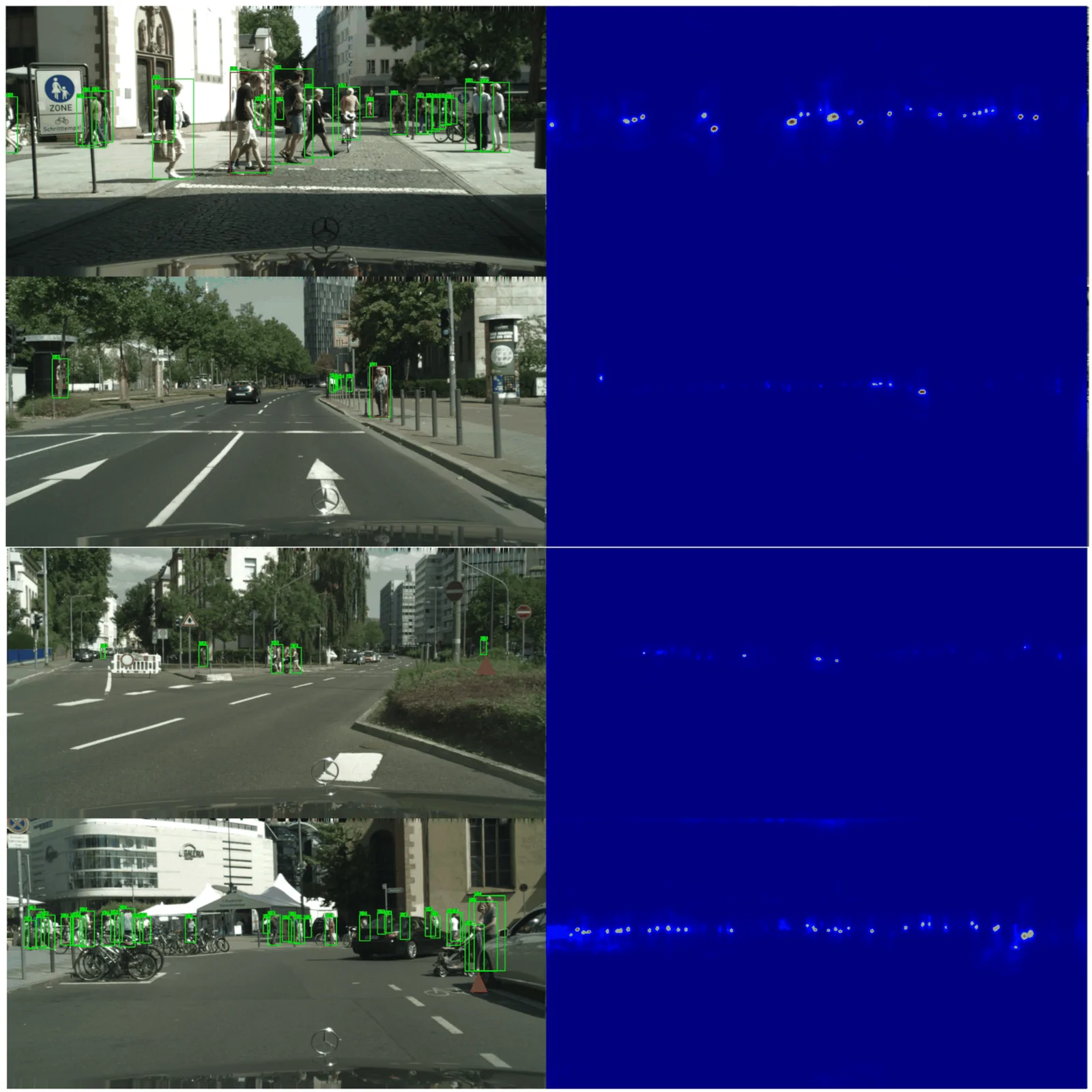
Failure cases on the CityPersons datasets. The red boxes represent false negatives in the images. Zoom in for more details.

This section presents a number of failure cases from Figure 8 and analyzes the limitations of the proposed approach. As illustrated in the upper figure on the left, the upper half of an instance is completely occluded. From the perspective of human vision, the number of instances can be determined by the number of legs. In the top right image, our model incorrectly identifies traffic signs as people in some instances. In our opinion, the primary cause of false positives is the visual similarity between traffic signs and pedestrians. As illustrated in the lower left panel, the occlusion of an object by true positives and the confusion of its head semantic information can result in the generation of false negatives. Since the response of an object in a Gaussian heatmap is normally distributed, the presence of positive samples with low response around the object presents a challenge for detection. In the bottom-right image, a circular object has the potential to be treated by the model as a person suffering from occlusion due to our enhanced head feature. Based on the study of failure cases, we believe that the lower part of pedestrians can not be ignored, but they are also the most vulnerable to occlusion, which seems to be a contradictory concept. We look forward to new work to address related issues.

To suppress false positives where rigid geometric structures (e.g., circular background objects) are misidentified as pedestrians, future work of our framework will incorporate shape-prior regularization to better distinguish flexible biological contours. Furthermore, integrating the infrared (thermal) modality into a multispectral framework represents a highly effective solution, as distinctive heat signatures can explicitly isolate human radiation profiles from inert, non-thermal geometric shapes. This multi-modal fusion, combined with spatial-semantic constraints, will substantially enhance the model’s discriminative robustness in complex environments.

While STG effectively handles typical occlusions, it has inherent limits due to its rigid spatial lattice. (1) Upper-Body Occlusion: Loss of upper anchor features deprives I2GI of reliable keys, as the fixed 5×9 grid cannot dynamically deform toward visible lower limbs. (2) Geometric False Positives: Traffic signs can falsely trigger top anchors, causing the rigid grid to force-align over background structures. (3) Non-Upright Poses: Severe pose deformations misalign anatomical parts with the rigid grid, causing axial cross-attention to aggregate feature noise.

## 5. Conclusions

This paper presents a novel framework for pedestrian detection in crowded scenes. It addresses the issue of correlation in global information interaction that is inherent in existing methods. In particular, a point feature screening mechanism based on a Gaussian heatmap is proposed to enhance feature representation by utilizing complementary gridpoint information and different gridpoint information from different instances. In terms of detection accuracy and running speed, our proposed method is superior to the majority of existing methods on the CityPersons and CrowdHuman datasets. It is capable of achieving a good balance between speed and accuracy.

Our future research will advance in three directions. First, we will transition from rigid gridpoints to deformable gridpoints combined with deformable attention, enabling adaptive tracking of dynamic human poses. Second, the framework will be extended to multimodal perception, leveraging thermal inputs to guide gridpoint visibility estimation in low-light environments. Finally, we aim to streamline the interaction networks via knowledge distillation or linear attention mechanisms to facilitate efficient deployment on edge computing devices.

## Figures and Tables

**Table 1:** Different gridpoints on CityPersons dataset (*MR*); **C** means coordinate of gridpoints. R and HO denote reasonable and heavy occlusion setting, respectively. The coordinates are aligned with the 5×9 lattice grid layout. The bold numbers indicate best performance in each column.

C	R	HO	C	R	HO	C	R	HO	C	R	HO	C	R	HO
(1,1)	12.26	36.39	(2,1)	10.72	35.83	(3,1)	10.33	35.07	(4,1)	11.07	35.55	(5,1)	12.09	37.29
(1,2)	11.63	35.76	(2,2)	11.09	34.72	(3,2)	10.51	**34.40**	(4,2)	11.49	35.61	(5,2)	13.01	37.74
(1,3)	13.73	35.74	(2,3)	11.21	35.30	(3,3)	10.30	34.80	(4,3)	10.95	37.15	(5,3)	14.11	36.81
(1,4)	12.76	38.23	(2,4)	10.81	35.72	(3,4)	9.40	34.48	(4,4)	11.76	35.17	(5,4)	12.76	46.79
(1,5)	12.24	37.17	(2,5)	10.32	37.65	(3,5)	**8.78**	35.03	(4,5)	9.77	35.53	(5,5)	9.92	37.83
(1,6)	12.16	38.18	(2,6)	10.27	37.17	(3,6)	9.29	36.87	(4,6)	11.29	37.53	(5,6)	12.24	38.08
(1,7)	11.39	37.03	(2,7)	10.80	36.92	(3,7)	9.36	36.35	(4,7)	10.47	37.79	(5,7)	12.89	38.33
(1,8)	12.24	36.78	(2,8)	10.27	36.07	(3,8)	9.63	36.29	(4,8)	10.25	35.98	(5,8)	12.60	37.70
(1,9)	14.58	39.10	(2,9)	13.78	38.18	(3,9)	11.79	38.15	(4,9)	13.16	41.40	(5,9)	14.27	40.07

**Table 2:** Effects of SAFF module and AIFI module on CityPersons datasets.

Primary	SAFF	AIFI	R	RS	HO	A
(3,2)			10.51	16.75	34.40	33.47
		✓	10.16	14.06	33.56	31.53
	✓	✓	**9.56**	14.03	31.67	31.10
(3,5)			8.78	11.90	35.03	30.97
		✓	8.33	10.65	34.13	29.22
	✓	✓	**8.14**	11.44	33.20	29.73

**Table 3:** Comparison with different auxiliary points with SAFF+AIFI+I2GI.

Primary	Auxiliary	CityPersons					WiderPerson	
		R	RS	HO	A		*MR*	AP
(3,5)	(3,2)	**7.41**	**10.46**	**32.05**	29.15		**40.74**	**90.22**
(3,5)	(3,3)	8.03	10.67	33.40	29.55		41.08	89.10
(3,5)	(3,4)	8.44	10.73	35.88	**29.06**		41.50	89.34
(3,5)	(3,6)	9.23	12.80	34.17	29.59		41.68	88.83
(3,5)	(3,7)	8.96	12.56	35.38	30.34		41.62	88.74
(3,5)	(3,8)	8.25	11.00	34.99	29.30		41.38	89.25

**Table 4:** Comparison with different modules with point (3,5) and auxiliary point (3,2).

Modules			CityPersons (*MR* ↓)				WiderPerson	
SAFF	AIFI	I2GI	R	RS	HO	A	*MR* (↓)	AP (↑)
			8.78	11.90	35.03	30.97	42.88	88.26
✓			9.02	12.42	34.20	29.61	42.57	88.33
	✓		8.33	10.65	34.13	29.22	42.30	88.58
✓	✓		8.14	11.44	33.20	29.73	41.82	88.62
		✓	7.87	10.74	34.00	29.17	41.03	88.79
✓	✓	✓	**7.41**	**10.46**	**32.05 **	**29.15 **	**40.74 **	**90.22 **

**Table 5:** Comparison with different gridpoints under different settings (SG and MG denote single and multiple gridpoints, SA and CA denote Self-Attention and Cross-Attention, respectively).

Methods	Gridpoints	R	RS	HO	A
SG Baseline	P(3,5)	9.61	12.51	36.15	31.50
SG Baseline	P(3,2)	12.12	18.66	35.50	34.10
MG Baseline	P(3,5)+P(3,2)	8.78	11.90	35.03	30.97
MG Baseline	P(3,2)+P(3,5)	10.51	16.75	34.40	33.47
MG+SA	P(3,5)+P(3,2)	8.33	10.65	34.13	29.22
MG+SA	P(3,2)+P(3,5)	9.19	13.90	38.87	30.96
MG+CA	P(3,5)+P(3,2)	7.87	10.74	34.00	29.17
MG+CA	P(3,2)+P(3,5)	8.75	12.10	33.92	29.14
MG+CA	P(3,5)+P(3,2)+P(2,5)	8.48	11.87	34.44	29.56
MG+CA	P(3,5)+P(3,2)+P(4,5)	8.26	10.99	34.02	29.90
MG+CA	P(3,2)+P(3,5)+P(2,2)	9.08	12.96	33.89	30.55
MG+CA	P(3,2)+P(3,5)+P(4,2)	9.34	13.34	33.75	30.76
Full STG	P(3,5)+P(3,2)	**7.41**	**10.46**	**32.05**	**29.15**

**Table 6:** Comparison with different backbones.

Backbone	Methods	R	RS	HO	A
ResNet18 [48]	Baseline	22.62%	36.89%	56.02%	49.38%
	Ours	17.26% (−5.36%)	27.62% (−9.27%)	50.08% (−5.94%)	43.15% (−6.23%)
DLA34 [49]	Baseline	8.78%	11.90%	35.03%	30.97%
	Ours	7.41% (−1.37%)	10.46% (−1.44%)	32.05% (−2.98%)	27.95% (−3.02%)

**Table 7:** Sensitivity ablation of threshold τ. Bold number means the best choice.

Threshold τ	0.30	0.35	0.40	0.45	0.50	0.55
Reasonable MR (%)	10.25	9.80	9.52	**9.35**	9.60	10.12

**Table 8:** Results on CityPersons validation datasets (*MR* (%)).

Methods	R	RS	HO
FCOS [50]	17.13%	25.66%	51.84%
FasterRCNN [51]	15.40%	25.60%	-
Repulsion loss [2]	13.20%	37.24%	56.90%
HBA-RCNN [9]	12.50%	15.68%	48.10%
DINO [28]	11.40%	-	43.10%
RE-YOLOv5 [52]	10.98%	-	40.31%
BetaRCNN [53]	10.60%	-	47.10%
NMS-loss [54]	10.08%	-	-
YOLOV5m [55]	10.18%	14.95%	37.55%
Sparse-RCNN [56]	10.00%	-	-
ACSP [57]	9.30%	-	46.30%
SOLIDER [31]	9.70%	-	39.40%
VLPD [58]	9.41%	10.93%	34.88%
GECC [59]	9.34%	11.90%	38.27%
FBCNet [60]	9.59%	12.11%	39.46%
MPLR-CapsNet [15]	9.10%	-	37.8%
OADB-Net [14]	8.70%	-	39.9%
VPKB [61]	7.60%	-	-
Ours	**7.41%**	**10.46%**	32.05%

**Table 9:** Results on Crowdhuman datasets. Bold numbers indicate the best performance in each column.

Methods	AP	*MR*
RetinaNet [62]	80.8%	63.3%
Adaptive NMS [10]	84.7%	47.7%
JED [12]	85.9%	53.5%
FPN + NMS [63]	84.9%	46.3%
FCOS [50]	86.8%	54.0%
IterDet [64]	84.4%	49.1%
Sparse RCNN [56]	91.3%	44.8%
OPL [32]	91.0%	44.9%
DiffusionDet [65]	**91.4%**	45.7%
Ours	85.2%	**44.2%**

**Table 10:** Results on WiderPerson datasets. Bold numbers indicate the best performance in each column.

Methods	Recall	AP	*MR*
Faster RCNN [51]	-	-	46.60 %
RetinaNet [62]	-	-	48.32 %
IterDet 1iter [64]	92.67%	89.49%	**40.35%**
PS-RCNN [66]	94.71%	89.96%	-
IterDet 2iter [64]	**97.15%**	**91.95%**	40.78%
Ours	93.51%	90.22%	40.74%

**Table 11:** Running Speed comparison. (NVIDIA RTX 3090 GPU, batch size 1, FP32, full pipeline latency including pre- and post-processing for image size of 1024×2048).

Methods	FCOS	Faster R-CNN	YOLOv5m	Ours
Speed (FPS)	15	15	20	16
FLOPS (G)	817.86	795.07	252.10	568.52
Params (M)	32.22	41.34	21.17	28.52

**Table 12:** Error metrics comparison on CrowdHuman validation set.

Method	AP (%)	MR (%)	FP/Image	FN/Image
Sparse R-CNN	91.10	44.30	1834/4370	22,080/4370
DiffusionDet	91.40	45.70	1686/4370	29,362/4370
STG	85.20	44.20	5452/4370	21,560/4370
STG (Threshold 0.45)			2490/4370	24,941/4370
STG (Threshold 0.55)			950/4370	39,525/4370

## Data Availability

The CityPersons (https://github.com/cvgroup-njust/CityPersons (accessed on 27 August 2026), CrowdHuman (https://www.crowdhuman.org/) (accessed on 27 August 2026), WiderPerson (http://www.cbsr.ia.ac.cn/users/sfzhang/WiderPerson/) (accessed on 27 August 2026), These three datasets are publicly available from their respective project websites.
